# Retraction Note: Break oily water emulsion during petroleum enhancing production processes using green approach for the synthesis of SnCuO@FeO nanocomposite from microorganisms

**DOI:** 10.1038/s41598-024-73161-w

**Published:** 2024-10-08

**Authors:** M. Hosny, Mahmoud F. Mubarak, H. S. El-Sheshtawy, R. Hosny

**Affiliations:** 1 Processes Development Department, Egyptian Petroleum Research Institute (EPRI), Nasr City, 11727 Cairo Egypt; 2 Petroleum Applications Department, Egyptian Petroleum Research Institute (EPRI), Nasr City, 11727 Cairo Egypt; 3 Production Department, Egyptian Petroleum Research Institute (EPRI), Nasr City, 11727 Cairo Egypt

Retraction of: *Scientific Reports* 10.1038/s41598-024-56495-3, published online 10 April 2024

The Editors have retracted this Article.

After publication concerns were raised about the spectra presented in three of the figures, namely:

- the SnCuO@FeO (green) FITR spectum in Fig. 3
- the SnCuO@FeO (1:1) (red) FITR spectrum in Fig. 4
- the SnCuO@FeO (blue) XRD spectrum in Fig. 5

In addition, reference 35 does not appear to be related to the study reported. The authors provided an explanation but this did not satisfactorily address the concerns. The Editors therefore no longer have confidence in the results and conclusions. M. Hosny, H. S. El-Sheshtawy and R. Hosny have not responded to correspondence from the Editors about this retraction. Mahmoud F. Mubarak has not explicitly stated if they agree or disagree with this retraction.

